# Associations of Depression, Anxiety, and Life Events With the Risk of Obstructive Sleep Apnea Evaluated by Berlin Questionnaire

**DOI:** 10.3389/fmed.2022.799792

**Published:** 2022-04-07

**Authors:** Xueru Duan, Murui Zheng, Wenjing Zhao, Jun Huang, Lixian Lao, Haiyi Li, Jiahai Lu, Weiqing Chen, Xudong Liu, Hai Deng

**Affiliations:** ^1^Department of Epidemiology, School of Public Health, Sun Yat-sen University, Guangzhou, China; ^2^School of Public Health, Guangdong Pharmaceutical University, Guangzhou, China; ^3^Guangzhou Center for Disease Control and Prevention, Guangzhou, China; ^4^School of Public Health and Emergency Management, Southern University of Science and Technology, Shenzhen, China; ^5^Department of Geriatrics, Institute of Geriatrics, Guangdong Provincial People's Hospital, Guangdong Academy of Medical Science, Guangzhou, China; ^6^Department of Cardiology, Guangdong Cardiovascular Institute, Guangdong Provincial People's Hospital, Guangdong Academy of Medical Science, Guangzhou, China

**Keywords:** depression, anxiety, obstructive sleep apnea, adverse life events, positive life events

## Abstract

**Background:**

Psychological problems are prevalent in the general population, and their impacts on sleep health deserve more attention. This study was to examine the associations of OSA risk with depression, anxiety, and life events in a Chinese population.

**Methods:**

A total of 10,287 subjects were selected from the Guangzhou Heart Study. Berlin Questionnaire (BQ) was used to ascertain the OSA. The Center for Epidemiologic Studies Depression Scale (CES-D) and Zung's self-rating anxiety scale (SAS) were used to define depression and anxiety. A self-designed questionnaire was used to assess life events. Odds ratio (OR) with 95% confidence interval (95% CI) was calculated by using the logistic regression model.

**Results:**

There were 1,366 subjects (13.28%) classified into the OSA group. After adjusting for potential confounders, subjects with anxiety (OR: 2.60, 95% CI: 1.63–4.04) and depression (OR: 1.91, 95% CI: 1.19–2.97) were more likely to have OSA. Subjects suffering from both anxiety and depression were associated with a 3.52-fold (95% CI: 1.88–6.31) risk of OSA. Every 1-unit increment of CES-D score and SAS index score was associated with 13% (95% CI: 1.11–1.15) and 4% (95% CI: 1.03–1.06) increased risk of OSA. Neither positive life events nor adverse life events were associated with OSA.

**Conclusions:**

The results indicate that depression and anxiety, especially co-occurrence of both greatly, were associated with an increased risk of OSA. Neither adverse life events nor positive life events were associated with any risk of OSA. Screening for interventions to prevent and manage OSA should pay more attention to depression and anxiety.

## Background

Obstructive sleep apnea (OSA), a relatively common sleep disorder in both general and specific disease-related populations (1), is characterized by recurrent episodes of a partial or complete collapse of the upper airway during sleep (2), with consequent oxygen desaturation, frequent arousals, and sleep fragmentation (3). The prevalence of OSA ranged from 9 to 38% in Europe and America (1) and was around 7% in Asia (4) and 24.2% in China (5). Approximately one billion adults aged 30–69 years worldwide are suffering from OSA and about 425 million required medical treatment (5). Moreover, OSA can further result in severe cardiovascular diseases and cognitive impairment (6–9).

Many factors including aging, male, obesity, snoring, and craniofacial and upper airway abnormalities were found to be associated with OSA occurrence (10, 11). Increasing literature is highlighting the critical effects of social and psychological problems on OSA-related sleep disorders (6, 12–14). According to Rezaeitalab et al. (15), anxiety and depression are the two most common comorbidities of sleep disorders and respiratory diseases. A meta-analysis found that the prevalence of OSA was 25.7% among patients with serious mental illness (16). Studies have found that anxiety and depression could disrupt sleep rhythm, and chronic disturbance of normal sleep could aggregate sleep apnea (13, 17–19). Depression and anxiety may occur simultaneously; it is found that about 85% of depressive patients have significant anxiety, and 90% of patients with anxiety disorder have depression (20). However, there lack of studies to examine the independent and joint effects of anxiety and depression on the occurrence of OSA.

Adverse life events, one of the major sources of social stress, were found to be related to the onset of a wide range of psychiatric disorders, such as depression, anxiety, and substance use (21). Some common adverse life events include divorce or separation, widowed, bereavement, serious illness of family members, serious natural disaster, unemployment, violence, and bankruptcy (21). Adverse life events are very common in the general population with most adults (60.7% of men and 51.2% of women) reporting having experienced at least one event in their lifetime (22). Tripathi et al. (23) found that early stressful life events might initiate and aggravate tissue inflammation, leading to deregulation in the hypothalamo-pituitary axis and an increase in serum levels of cortisol and C-reactive protein, and in turn finally result in the development of OSA. Nevertheless, the association between OSA risk and life events is unclear.

Therefore, this study aimed to investigate the associations of depression, anxiety, and life events with OSA occurrence.

## Materials and Methods

### Setting and Subjects

**Figure 1:**
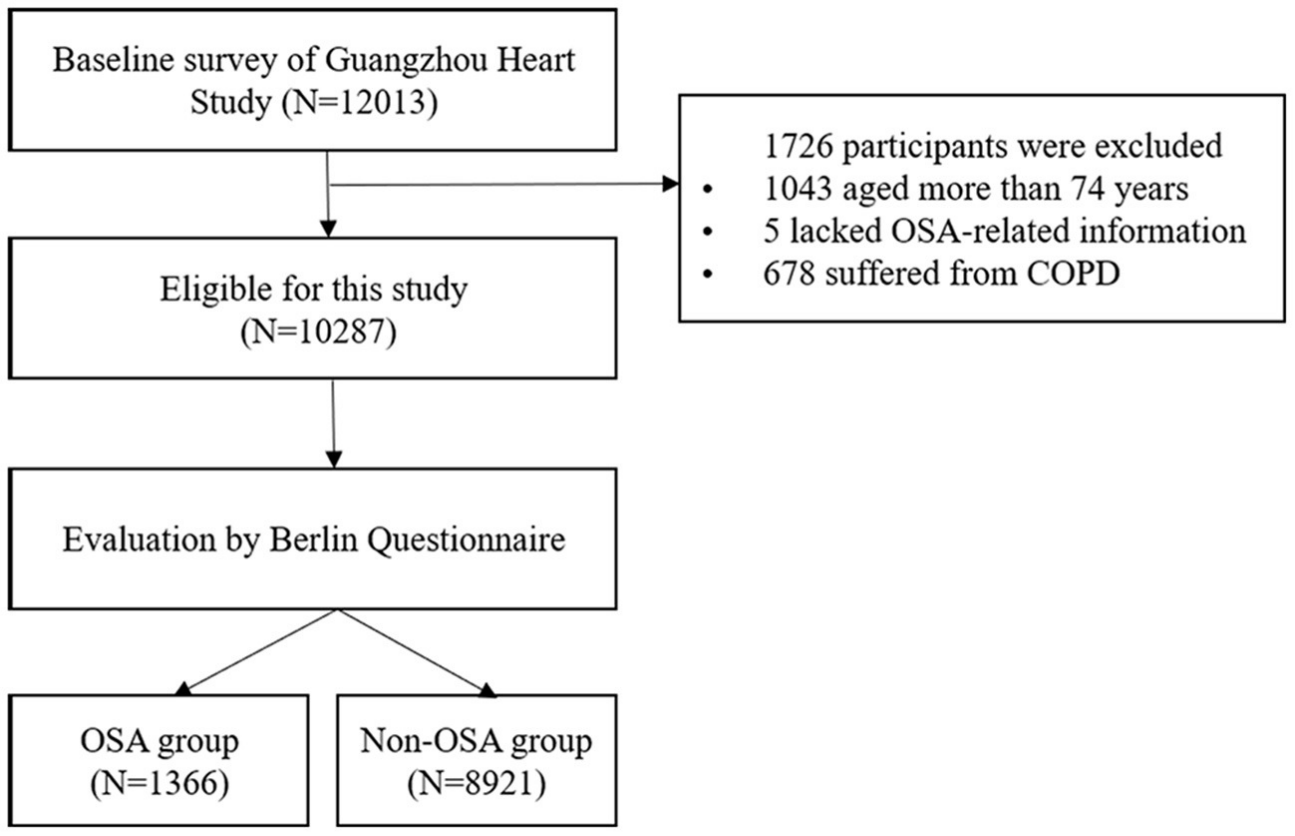
Flow chart for the selection process of subjects.

Subjects in this cross-sectional study were selected from the baseline survey of an ongoing prospective longitudinal study-the Guangzhou Heart Study. A detailed description of the cohort has been presented in our previous reports (24, 25). In brief, a total of 12,013 permanent residents aged ≥35 years accomplished the baseline survey during the year 2015-2017. In this study, 1,726 participants were excluded for the age of more than 74 years (1,043 participants), incomplete OSA-related information (5 participants), or suffering from a chronic obstructive pulmonary disease (678 participants). Finally, a total of 10,287 subjects were included in the following analysis. The flow chart for the selection process of subjects can be seen in Figure 1. This study was approved by the Ethical Committee of Guangdong Provincial People's Hospital and by the Ethical Review Committee for Biomedical Research, School of Public Health, Sun Yat-sen University. The study was performed in line with the Declaration of Helsinki and all participants provided informed consent.

### OSA Ascertainment

The risk of OSA was assessed with Berlin Questionnaire (BQ) (26). The 10-item questions comprise three categories: frequency and intensity of snoring (category 1, items 1–5), frequency of daytime sleepiness or fatigue (category 2, items 6–9), body mass index (BMI), and hypertension (category 3, item 10). The first and second categories were assessed as positive if persistent symptoms (>3 to 4 times/week) were reported, while category 3 was positive if there was a history of hypertension or a BMI of higher than 30 kg/m^2^. If two or more categories were positive, the participant was considered at high risk of OSA and classified into the OSA group, otherwise at low risk of OSA and classified into the non-OSA group (27).

### Depression, Anxiety, and Life Events Assessment

Depression and anxiety were measured using the Center for Epidemiologic Studies Depression Scale (CES-D) (28) and Zung's self-rating anxiety scale (SAS) (29), both of which have been demonstrated to have credible internal consistency and adequate test-retest repeatability in the Chinese population (29, 30). The CES-D scale contains 20 items. Each participant was asked to answer the frequency of feelings or behaviors for each item over the past week. Ratings were based on a 4-point scale from 0 (rarely or none of the time) to 3 (most or all the time); the items of 4, 8, 12, and 16 are reverse-scored; the scores for all items are added to get the total score, that is the CES-D score (28). The CES-D score ranges from 0 to 60 and a participant with the score of ≥16 was defined as having depression and otherwise having no depression (28, 31).

The SAS is a 20-item scale, adopting a 4-point scale ranging from 1 (none, or a little of the time) to 4 (most, or all the time). Fifteen items presented adverse experiences, and 5 items presented positive experiences with reverse scoring. Each participant was asked to select the frequency of each item and the total score was calculated by adding the score of each item. The raw total score ranged from 20 to 80, and then the raw total score was converted to an index score by dividing the sum of the raw score by 80 and multiplying by 100 (29). Therefore, the SAS index score ranged from 25 to 100 and a participant with the SAS index score of ≥45 was judged as having anxiety, otherwise as having no anxiety (29, 32).

Considering that depression and anxiety often coexist (33) and the depression and anxiety comorbidity may lead to higher severity of illness (34), we categorized all participants into three groups (Neither, One, Both) according to the status of the combination of depression and anxiety. Participants in the Neither group means they had neither depression nor anxiety; participants in the One group means they had either depression or anxiety; participants in the Both group means they had both depression and anxiety. Neither group was taken as the reference group for further analysis. In addition, the CES-D score and SAS index score were converted from continuous variables into categorical variables based on cut-off points of tertiles of scores.

Life event was defined as a social experience or change with a specific onset and course that has a psychological impact on a person (21). A self-designed 10-item structured questionnaire was used to collect the information of the ten life events commonly occurring in daily life (Supplementary Figure 1). The questionnaire was examined twice with 2 month-intervals among 180 local adults and found to have moderate to good reliability and reproducibility (unpublished). This questionnaire contained eight items of adverse life events and two items of positive life events. Eight adverse life events included divorce or separation, widowed, bereavement, serious illness of family members, serious natural disaster, unemployment or lay-off, suffering from violence, and bankruptcy or large property loss; two positive life events included winning the lottery or a windfall, and joyful events. Each participant was asked to answer whether he or she had experienced a certain life event during the past year; if the response was “yes,” it meant that he or she had experienced the corresponding event, otherwise not. For the adverse life events, a point of 0 was assigned to each item if the response was “no” and a point of 1 was assigned if the response was “yes;” for the positive life events, a point of 0 was assigned to each item if the response was “no” and a point of 1 was assigned if the response was “yes.” Then, we calculated the total scores of adverse life events and positive life events, respectively; if the total score of adverse life events or positive life events of a participant was non-zero, this participant was deemed to have experienced an adverse or positive life event, otherwise not experienced. Some studies have investigated the overall effect of both adverse and positive life events (35, 36), we also calculated the life event score for each participant by summing each score of all life events, and divided the participants into three groups with zero as the cut-point: <0 (means that the number of positive life events exposure exceeds the number of adverse life event exposure), 0 (means that the number of positive life events exposure equals to the number of adverse life event exposure, or means that the participant dose not expose to any life events), >0 (means that the number of adverse life events exposure exceeds the number of positive life event exposure).

### Potential Confounding Factors

A self-administered questionnaire was used to collect demographic and lifestyle information, including age (years), sex (male, female), education (primary school or lower, junior high school, senior high school, and college or above), and marital status (married and others), smoking (non-smoker, ex-smoker, and current smoker), alcohol drinking (never, occasion and frequent), fruit intake (≥once/day, < once/day), vegetable intake (≥once/day, < once/day) and leisure-time physical activity (LTPA). The total volume of leisure-time physical activity was the sum of the volume of each type of leisure-time physical activity, which was obtained by multiplying the intensity of the physical activity by its duration and by its frequency. The physician-diagnosed disease of hypertension (yes, no), dyslipidemia (yes, no), cardiovascular disease (yes, no) was required to report. Cardiovascular disease included any myocardial infarction, stroke, valvular heart disease, heart failure, and atrial fibrillation. Physical measurements, including height, weight, waist circumference, hip circumference, and blood pressure, were performed in line with standard instruments and protocols. Body mass index was calculated as weight divided by height squared (kg/m^2^). The waist-hip ratio was calculated by dividing waist circumference (cm) by hip circumference (cm).

### Statistical Analyses

Shapiro-Wilk test was used to examine the normality; then the chi-squared test, *t*-test, and Wilcoxon rank-sum test were used to examine the distribution of categorical and continuous variables between the OSA group and the non-OSA group. Cochran-Armitage trend test was used for the distribution of a combination of depression and anxiety. We established three models: model 1 was not adjusted for any variable; model 2 was adjusted for age, sex, education, marital status, waist-hip ratio, leisure-time physical activity, smoking, alcohol drinking, fruit intake, vegetables intake, and dyslipidemia; model 3 was adjusted for body mass index as well as the covariables adjusted in the model 2. All confounders were selected based on the directed acyclic graph (DAG) model of OSA and the minimal sufficient adjustment sets for estimating the direct effect of anxiety, depression, life events on OSA were adopted (Supplementary Figure 2). Unadjusted and adjusted odds ratios (OR) with 95% confidence intervals (CI) were estimated by using logistic regression. Given the depressive and anxiety symptoms often co-occurred, we estimated the association of combined exposure of depression and anxiety with OSA risk. To examine the stability and robustness of the results, we did the sensitivity analysis by excluding participants aged 60 years or above and excluding those with cardiovascular disease. In addition, overweight and obesity are related to anxiety and depression, so we conducted a sensitivity analysis by using the OSA subitems [snoring (category 1), daytime sleepiness (category 2), obesity (category 3, defined as BMI more than 30 kg/m^2^), and hypertension (category 3)] as the outcomes to eliminate the potential bias. Statistical analysis was conducted by using R (version 3.6.3). All tests were two-tailed and a *P-*value of <0.05 was considered statistically significant.

## Results

A total of 10,287 participants were included in this study, of which 8,921 (86.72%) were classified into the non-OSA group and 1,366 (13.28%) into the OSA group (Table 1). There were 153 participants having depression, 143 having anxiety, and 70 having both depression and anxiety. In comparison with the non-OSA group, the OSA group has a higher proportion of patients with anxiety and positive life events (*P* <0.05). There was no significant difference in the occurrence of depression and adverse life events between the non-OSA group and the OSA group (*P* > 0.05).

**Table 1:** Baseline characteristics of the participants by OSA.

**Characteristic**	**Non-OSA group (*N* = 8,921)**	**OSA group (*N* = 1,366)**	***P-*value**
Age (years), mean (S.D.)	56.02 (10.01)	57.89 (9.17)	<0.001^a^
Leisure-time physical activity, MET-h/week, median (IQR)	35.70 (17.90, 59.20)	33.60 (12.43, 57.10)	<0.001^d^
Body mass index, kg/m^2^, mean (S.D.)	23.61 (3.24)	26.74 (3.97)	<0.001^a^
Waist-hip ratio, mean (S.D.)	0.88 (0.07)	0.92 (0.07)	<0.001^a^
Age, *N* (%)			<0.001^b^
35–54	3,922 (43.96)	501 (36.68)	
55–74	4,999 (56.04)	865 (63.32)	
Sex, *N* (%)			<0.001^b^
Male	2,797 (31.35)	712 (52.12)	
Female	6,124 (68.65)	654 (48.87)	
Education, *N* (%)			0.003^b^
Primary school or less	3,185 (35.70)	489 (35.80)	
Junior high school	2,259 (25.32)	342 (25.04)	
Senior high school	2,261 (25.35)	392 (28.70)	
Junior college or higher	1,216 (13.63)	143 (10.46)	
Marital status, *N* (%)			<0.001^b^
Married	7,801 (87.45)	1,251 (91.58)	
Others	1,120 (12.55)	115 (8.42)	
Smoke, *N* (%)			<0.001^c^
Non-smoker	7,218 (80.91)	945 (69.18)	
Ex-smoker	396 (4.44)	127 (9.30)	
Current-smoker	1,307 (14.65)	294 (21.52)	
Alcohol drinking, *N* (%)			<0.001^c^
Never	7,043 (78.95)	962 (70.43)	
Occasion	1,394 (15.63)	265 (19.40)	
Frequent	484 (5.42)	139 (10.17)	
Fruit intake, *N* (%)			<0.001^b^
≥Once/day	5,778 (64.77)	799 (58.49)	
< Once/day	3,143 (35.23)	567 (41.51)	
Vegetable intake, N (%)			0.310^b^
≥Once/day	8,608 (96.49)	1,310 (95.90)	
< Once/day	313 (3.51)	56 (4.10)	
Hypertension, *N* (%)			<0.001^b^
No	7,024 (78.74)	240 (17.57)	
Yes	1,897 (21.26)	1,126 (82.43)	
Cardiovascular disease, *N* (%)			<0.001^b^
No	442 (4.95)	112 (8.20)	
Yes	8,479 (95.05)	1,254 (91.80)	
Dyslipidemia, *N* (%)			<0.001^b^
No	2,755 (30.88)	338 (24.74)	
Yes	6,166 (69.12)	1,028 (75.26)	
Depression, *N* (%)			0.138^b^
No	8,975 (98.59)	1,339 (98.02)	
Yes	126 (1.41)	27 (1.98)	
Anxiety, *N* (%)			0.018^b^
No	8,807 (98.72)	1,337 (97.87)	
Yes	114 (1.28)	29 (2.12)	
Combination of depression and anxiety			0.017^c^
Neither	8,733 (97.89)	1,328 (97.22)	
One	136 (1.52)	20 (1.46)	
Both	52 (0.58)	18 (1.32)	
Adverse life event, *N* (%)			0.857^b^
No	8,061 (90.36)	1,237 (90.56)	
Yes	860 (9.64)	129 (9.44)	
Positive life event, *N* (%)			0.047^b^
No	8,264 (92.64)	1,265 (91.07)	
Yes	657 (7.36)	124 (8.93)	

a *P-value for t-test*.

b *P-value for chi-square test*.

c *P-value for Cochran-Armitage trend test*.

d *P-value for Wilcox rank sum test*.

The means of age, BMI and waist-hip ratio were all larger in the OSA group than those in the non-OSA group (all *P* < 0.05), whereas, the mean of the volume of leisure-time physical activity was higher in the non-OSA group (*P* < 0.05). Besides, more participants in the non-OSA group than in the OSA group were non-smoker, non-drinker, ate fruits at least once per day, ate vegetables at least once per day, and did not have hypertension, dyslipidemia, or cardiovascular disease (all *P* < 0.05).

Participants suffering from anxiety (OR: 2.60, 95% CI: 1.63–4.04) and depression (OR: 1.91, 95% CI: 1.19–2.97) were associated with an increased risk of OSA after considering potential confounders (Table 2). In comparison to participants with neither depression nor anxiety, those who only suffered from depression or only suffered from anxiety were associated with 1.35-fold (95% CI: 0.80–2.19) risk of OSA, those suffering from both depression and anxiety were associated with 3.52-fold (95% CI: 1.88–6.31) risk of OSA; a significant exposure-response trend was also observed (*P* < 0.001). Neither adverse life events nor positive life events were associated with any risk of OSA. The life event score was not associated with the risk of OSA, even every 1-unit increment of life event score did not reach a significant result.

**Table 2:** Association of depression, anxiety, and life events with OSA risk.

	**N^a^**		**Effect estimates**		
	**Non-OSA group**	**OSA group**	**Unadjusted OR (95% CI)**	**Adjusted OR (95% CI)^b^**	**Adjusted OR (95% CI)^c^**
Depression
No	8,975	1,339	1.00	1.00	1.00
Yes	126	27	1.41 (0.91, 2.11)	1.60 (1.02, 2.44)	1.91 (1.19, 2.97)
Anxiety
No	8,807	1,337	1.00	1.00	1.00
Yes	114	29	1.68 (1.09, 2.49)	2.11 (1.35, 3.20)	2.60 (1.63, 4.04)
Combination of depression and anxiety
Neither	8,733	1,328	1.00	1.00	1.00
One	136	20	0.97 (0.59, 1.51)	1.18 (0.71, 1.87)	1.35 (0.80, 2.19)
Both	52	18	2.28 (1.29, 3.83)	2.70 (1.49, 4.70)	3.52 (1.88, 6.31)
*P* for trend			0.017	0.018	<0.001
Adverse life event
No	8,061	1,237	1.00	1.00	1.00
Yes	860	129	0.98 (0.80, 1.18)	1.06 (0.87, 1.30)	1.08 (0.87, 1.33)
Positive life event					
No	8,264	1,244	1.00	1.00	1.00
Yes	657	122	1.23 (0.99, 1.50)	1.27 (1.03, 1.56)	1.24 (0.99, 1.54)
Life event score
<0	589	104	1.00	1.00	1.00
0	7,529	1,147	0.86 (0.70, 1.08)	0.83 (0.66, 1.04)	0.84 (0.67, 1.07)
>0	803	115	0.81 (0.61, 1.08)	0.85 (0.63, 1.14)	0.89 (0.65, 1.21)
*P* for trend			0.165	<0.001	<0.001
Every 1-unit increment			0.92 (0.80, 1.04)	0.95 (0.82, 1.09)	0.96 (0.83, 1.11)

a *N represents sample size for the non-OSA group or for the OSA group; OSA represents obstructive sleep apnea*.

b *Adjustment for age, sex, education, marital status, waist-hip ratio, leisure-time physical activity, smoking, alcohol drinking, fruit intake, vegetables intake, and dyslipidemia*.

c *Additional adjustment for body mass index*.

The risk of OSA increased with the increment of the CES-D score and SAS index score (Table 3). Every 1-unit increment of CES-D score was associated with a 13% increased risk of OSA (OR: 1.13, 95% CI: 1.11–1.15) and every 1-unit increment of SAS index score was associated with a 4% increased risk of OSA (OR: 1.04, 95% CI: 1.03–1.06) after adjustment for confounders; when comparing with the lowest tertile of the score, the highest tertile of the CES-D score and SAS index score were associated with 27% (95% CI: 1.10–1.47, *P*_−trend_ <0.001) and 40% (95% CI: 1.21–1.62, *P*_−trend_ <0.001) increased risk of OSA after considering potential confounders.

**Table 3:** Association of CES-D score and SAS index score with OSA risk.

	**N^a^**		**Effect estimates**		
	**Non-OSA group**	**OSA group**	**Unadjusted OR (95% CI)**	**Adjusted OR (95% CI)^b^**	**Adjusted OR (95% CI)^c^**
CES-D score
Tertile 1	3,694	565	1.00	1.00	1.00
Tertile 2	2,471	361	0.96 (0.83, 1.10)	1.06 (0.91, 1.22)	1.15 (0.99, 1.34)
Tertile 3	2,756	440	1.04 (0.91, 1.19)	1.15 (1.00, 1.32)	1.27 (1.10, 1.47)
*P* for trend			0.579	<0.001	<0.001
Every 1-unit increment			1.13 (1.11, 1.14)	1.13 (1.11, 1.15)	1.13 (1.11, 1.15)
SAS index score
Tertile 1	3,591	513	1.00	1.00	1.00
Tertile 2	2,595	402	1.08 (0.94, 1.25)	1.16 (1.01, 1.34)	1.19 (1.02, 1.38)
Tertile 3	2,735	451	1.15 (1.01, 1.32)	1.30 (1.12, 1.49)	1.40 (1.21, 1.62)
*P* for trend			0.038	<0.001	<0.001
Every 1-unit increment			1.02 (1.01, 1.03)	1.03 (1.02, 1.05)	1.04 (1.03, 1.06)

a *N represents sample size for the non-OSA group or for the OSA group; OSA represents obstructive sleep apnea; CES-D score represents the Center for Epidemiologic Studies Depression Scale score; SAS index score represents index score from Zung's self-rating anxiety scale*.

b *Adjustment for age, sex, education, marital status, waist-hip ratio, leisure-time physical activity, smoking, alcohol drinking, fruit intake, vegetables intake, and dyslipidemia*.

c *Additional adjustment for body mass index*.

In sensitivity analysis, repeated analyses were conducted after excluding the subjects aged 60 years or above and similar consistent results were obtained (Supplementary Tables 1, 2). We also did the analysis after excluding participants with cardiovascular disease, and similar results were obtained except for depression (Supplementary Table 3); however, when comparing the highest with lowest tertiles, CES-D score was associated with increased OSA risk and every 1-unit increment of the CES-D score was associated with 1.12-fold (95% CI: 1.10–1.14) risk of OSA after adjusting for potential confounders (Supplementary Table 4). In the sensitivity analyses by using OSA subitems as outcomes, depression, anxiety, adverse life events, and positive life events were all not associated with obesity and hypertension. However, depression and anxiety, especially their coexistence, were associated with an increased risk of snoring and daytime sleepiness; exposure to adverse life events was associated with a 34% increased risk of daytime sleepiness (Supplementary Tables 5–8).

## Discussion

The results from this study manifest that the presence of anxiety and depression was associated with an increased risk of OSA and the coexistence of depression and anxiety had a more significant adverse effect on OSA risk. Neither adverse life event nor positive life event was associated with OSA risk.

In this study, participants with anxiety were 2.60 times more likely to suffer from OSA than those without anxiety after adjustment for all potential confounders. This was consistent with the results from a lifestyle intervention that the persistence of anxiety was independently associated with elevated levels of sleep-disordered breathing (37). Moreover, we also observed that every 1-unit increment of SAS index score was associated with a 4% increased risk of OSA, indicating that the impact of anxious mood accumulation on OSA may be progressive. The possible mechanism may be due to that anxiety could influence several brain regions, including ventral medial prefrontal, cingulate, parietal, and insular cortices, and the uncus of the hippocampal formation, extending to the amygdala (38). These regions oversaw the regulation of respiratory control, fear emotion, cognition, sensory and motor action (38). Besides, anxiety-like behavior can cause stress, which in turn can raise the level of cortisol and then act on glucocorticoid (GC) receptors (39, 40). GC-induced neurotoxicity can damage the hippocampus, amygdala, and prefrontal cortex accompanying repeated episodes of apnea and anxiety (41). After a single GC exposure, hippocampal dendrites showed reversible damage, and repeated exposure may elicit injury. The hippocampal loss was also reported to be correlated with the severity of OSA (42). Both animal and human researches corroborated that physiological, psychological, and metabolic consequences of increased sympathetic nerve tone associated with anxiety and inflammatory mediators can contribute to the pathogenesis of OSA (39, 43). The Lifelines study with 54,326 participants has demonstrated that anxiety disorders were associated with higher serum C-reactive protein (CRP) levels after adjusting for all covariates (43). Persistent anxiety disrupted autonomic nervous system functions and provoked low-grade inflammation, and pronounced pro-inflammatory states have also been directly linked to both anxiety and OSA (37).

Depression was observed to improve the risk of OSA in this study, which was consistent with results from a population-based longitudinal study among Taiwanese (18). Subjects might suffer from age-related diseases with aging, and the latter was reported to have a relationship with OSA (44–47). In this study, we yielded similar results that depression was associated with an increased risk of OSA after excluding subjects aged 60 years or more. The association of OSA risk with depression did not reach statistical significance after excluding participants with cardiovascular disease. This may be due to that most of the subjects with cardiovascular diseases aged more than 60 years old and only a few subjects left after excluding subjects with cardiovascular diseases. However, our study found that every 1-unit increment of CES-D score was associated with a 13% increased risk of OSA, indicating the even very mild symptom of depression could increase the risk of adverse health consequences. The possible effects of depression on OSA might be due to that depression can lead to notably difficulties falling asleep, frequent awakenings during the night and early morning awakenings, a shortened rapid eye movement latency as well as non-refreshing sleep (48). In addition, depression and anxiety could increase the risk of snoring and daytime sleepiness, which was consistent with previous studies (49–51). A population-based 10-year follow-up study found that anxiety and depression were the most important factors for predicting incidents of excessive daytime sleepiness (49). Gould et al. found that affective anxiety symptoms and depressive symptoms have independent associations with sleep disturbance (50). A Chinese cohort study of 0.5 million adults demonstrated the significant positive association between depression and daytime napping, as well as daytime dysfunction and snoring (51).

We found that the impact of both depression and anxiety exposure on OSA risk was much more significant than that of the single exposure. Although depression and anxiety have essentially been seen as distinct conditions, the two disorders were not mutually exclusive and often coexist to varying degrees in the same patient possibly due to the overlapped risk factors (33). In this study, nearly half of depressed participants suffered from anxiety simultaneously. Patients who have depression and anxiety comorbidity tend to have higher severity of illness and significantly greater impairment in work functioning, psychosocial functioning, and quality of life than patients not suffering from comorbidity (34).

Positive life event exposure was not observed to be related to the OSA risk, which was inconsistent with reports by Tripathi et al. (23). Studies have found that adverse life events could induce acute or chronic stress, which might lead to deregulation in the hypothalamo-pituitary axis and eventually accelerated the occurrence of OSA by increasing serum levels of cortisol and CRP (23). Vahtera et al. (52) found that exposure to adverse life events was strongly associated with sleep disturbances. However, no association between adverse life events and OSA risk was found in this study. This might be due to the limited number of adverse life events collected in our study, which might not well reflect the whole impact of multiple dimensional life events on OSA. However, the sensitivity analysis showed that exposure to adverse life events was associated with a 34% increased risk of daytime sleepiness, which indicated that adverse life events might impair nighttime sleep and leading to daytime sleepiness.

Given the significant discrepancy in life experience and physical functioning between middle-aged and elderly adults (53, 54), we excluded older adults aged more than 60 years for sensitivity analysis and obtained consistent results. Older people experienced retirement, relocation to more appropriate housing, and others; their lifestyles might be changed accordingly (54). Studies have demonstrated that exposure to adverse life events could have an especially erosive effect on older adults than younger and middle-aged adults (53). In addition, Geriatric syndrome can develop as the elderly age (55), and obstructive sleep apnea of geriatric patients was generally associated with sarcopenia, poor sleep quality, cognitive dysfunction, and nocturia different from the classic syndrome of obstructive sleep apnea of middle-age (56). Moreover, there are also many common risk factors between OSA and chronic diseases such as cardiovascular disease, the sensitivity analysis which excluded older adults aged more than 60 years or excluded subjects with cardiovascular disease could rule out potential bias caused by aging and other comorbidities. Our four sensitivity analyses implied that depression and anxiety were associated with the increased risk of snoring and daytime sleepiness but not obesity or hypertension. The effect of depression and anxiety on OSA was not mediated or confounded by obesity, which improved the reliability of this study greatly.

This study has several strengths. First, we used international standardized questionnaires to ascertain OSA, depression, and anxiety, which help to minimize information bias and benefit the comparisons with counterpart studies. Second, selection bias was minimized by using the multi-stage sampling method, which could to some degree make sure that participants in this study had good representativeness. Third, we adjusted as many as covariates to control the confounders using the multivariate model. Fourth, we performed several sensitivity analyses to examine the stability of our results, and the consistent results indicate the robustness of our results.

The limitations of this study are as follows. First, the prevalence of depression (1.49%) and anxiety (1.39%) in this study was lower than those in other studies which used the same instruments to assess depression and anxiety (16). Because of social stigma and the diverse nature of psychotic symptoms in China (57), the prevalence of depression and anxiety as obtained by a face-to-face interview in community surveys might be underestimated. Nevertheless, we found that OSA risk increased with the increment of the CES-D score and the SAS index score, indicating that the risk caused by depression and anxiety on OSA was credible to a large degree. Second, the diagnosis of OSA was based on Berlin Questionnaire, which was mostly used as a clinical screening test and epidemiological tool; we could not classify the severity of OSA due to the lack of polysomnography. Third, causal inference can not be made due to the restriction of cross-sectional analysis, so it is essential to be alert to reverse causality. However, our results were comparable to other studies (18, 23, 33, 37), which indicating that to some degree our results were credible. In the coming future, we will solve these problems with a prospective longitudinal design and more precise measurements of exposure and outcome.

## Conclusions

The results indicate that depression and anxiety, especially co-occurrence of both greatly, were associated with an increased risk of OSA. Neither adverse life events nor positive life events were associated with any risk of OSA. Screening for interventions to prevent and manage OSA should pay more attention to depression and anxiety.

## Data Availability Statement

The raw data supporting the conclusions of this article will be available from the corresponding author upon request. A proposal with description of study objectives and statistical analysis plan will be needed for evaluation of the reasonability of requests if someone requests data sharing.

## Ethics Statement

The studies involving human participants were reviewed and approved by the Ethical Committee of Guangdong Provincial People's Hospital and the Ethical Review Committee for Biomedical Research, School of Public Health, Sun Yat-sen University. The patients/participants provided their written informed consent to participate in this study.

## Author Contributions

XL and HD conceived and designed the study. XD, MZ, JH, LL, and HL collected the data. XD analyzed the data. XD, MZ, and JH drafted the manuscript. LL, HL, WZ, JL, WC, HD, and XL reviewed and edited the manuscript. All authors read and approved the final manuscript.

## Funding

This work was supported by the National Key R&D Program of China (Nos. 2018YFC1312502 and 2018YFE0208000), the Guangdong Provincial Key R&D Program (No. 2019B020230004), the Guangdong Basic and Applied Basic Research Foundation (No. 2019A1515011599), and the Science and Technology Program of Guangzhou City (No. 202102080404). The founder had no role in the design, analysis, or writing of this manuscript.

## Conflict of Interest

The authors declare that the research was conducted in the absence of any commercial or financial relationships that could be construed as a potential conflict of interest.

## Publisher's Note

All claims expressed in this article are solely those of the authors and do not necessarily represent those of their affiliated organizations, or those of the publisher, the editors and the reviewers. Any product that may be evaluated in this article, or claim that may be made by its manufacturer, is not guaranteed or endorsed by the publisher.

## Abbreviations

OSA: obstructive sleep apnea
BQ: Berlin Questionnaire
CES-D: The Center for Epidemiologic Studies Depression Scale
SAS: Zung's self-rating anxiety scale
OR: Odds ratio
CI: confidence interval
BMI: body mass index
GC: glucocorticoid
CRP: C-reactive protein
LTPA: leisure-time physical activity
DAG: directed acyclic graph.

## References

[1] Senaratna CV Perret JL Lodge CJ Lowe AJ Campbell BE Matheson MC. Prevalence of obstructive sleep Apnea in the general population: a systematic review. Sleep Med Rev. (2017) 34:70–81. 10.1016/j.smrv.2016.07.002 27568340

[2] Punjabi NM. The epidemiology of adult obstructive sleep Apnea. Proc Am Thorac Soc. (2008) 5:136–43. 10.1513/pats.200709-155MG 18250205 PMC2645248

[3] Sunwoo JS Hwangbo Y Kim WJ Chu MK Yun CH Yang KI. Prevalence, sleep characteristics, and comorbidities in a population at high risk for obstructive sleep apnea: a nationwide questionnaire study in South Korea. PLoS ONE. (2018) 13:e0193549. 10.1371/journal.pone.0193549 29489913 PMC5831105

[4] Mirrakhimov AE Sooronbaev T Mirrakhimov EM. Prevalence of obstructive sleep apnea in Asian adults: a systematic review of the literature. BMC Pulm Med. (2013) 13:10. 10.1186/1471-2466-13-10 23433391 PMC3585751

[5] Benjafield AV Ayas NT Eastwood PR Heinzer R Ip MSM Morrell MJ. Estimation of the global prevalence and burden of obstructive sleep Apnoea: a literature-based analysis. Lancet Respir Med. (2019) 7:687–98. 10.1016/S2213-2600(19)30198-5 31300334 PMC7007763

[6] Garbarino S Bardwell WA Guglielmi O Chiorri C Bonanni E Magnavita N. Association of anxiety and depression in obstructive sleep Apnea patients: a systematic review and meta-analysis. Behav Sleep Med. (2020) 18:35–57. 10.1080/15402002.2018.1545649 30453780

[7] Young T Skatrud J Peppard PE. Risk factors for obstructive sleep Apnea in adults. Jama. (2004) 291:2013–6. 10.1001/jama.291.16.2013 15113821

[8] Gao C Guo J Gong T-T Lv J-L Li X-Y Liu F-H. Sleep duration/quality with health outcomes: an umbrella review of meta-analyses of prospective studies. Front Med. (2022) 8:813943. 10.3389/fmed.2021.813943 35127769 PMC8811149

[9] Steardo L de Filippis R Carbone EA Segura-Garcia C Verkhratsky A De Fazio P. Sleep disturbance in bipolar disorder: neuroglia and circadian rhythms. Front Psychiatry. (2019) 10:501. 10.3389/fpsyt.2019.00501 31379620 PMC6656854

[10] Xu Z Wu Y Tai J Feng G Ge W Zheng L. Risk factors of obstructive sleep Apnea syndrome in children. J Otolaryngol Head Neck Surg. (2020) 49:11. 10.1186/s40463-020-0404-1 32131901 PMC7057627

[11] Zhou X Lu Q Li S Pu Z Gao F Zhou B. Risk factors associated with the severity of obstructive sleep Apnea syndrome among adults. Sci Rep. (2020) 10:13508. 10.1038/s41598-020-70286-6 32782271 PMC7421897

[12] BaHammam AS Kendzerska T Gupta R Ramasubramanian C Neubauer DN Narasimhan M. Comorbid depression in obstructive sleep Apnea: an under-recognized association. Sleep Breath. (2016) 20:447–56. 10.1007/s11325-015-1223-x 26156890

[13] Andrews JG Oei TP. The roles of depression and anxiety in the understanding and treatment of obstructive sleep Apnea syndrome. Clin Psychol Rev. (2004) 24:1031–49. 10.1016/j.cpr.2004.08.002 15533283

[14] Okada I Miyata S Iwamoto K Fujishiro H Noda A Ozaki N. Prevalence of obstructive sleep Apnea as assessed by polysomnography in psychiatric patients with sleep-related problems. Sleep Breath. (2022). 10.1007/s11325-022-02566-6. [Epub ahead of print]. 35029795

[15] Rezaeitalab F Moharrari F Saberi S Asadpour H Rezaeetalab F. The correlation of anxiety and depression with obstructive sleep Apnea syndrome. J Res Med Sci. (2014) 19:205–10. 24949026 PMC4061640

[16] Stubbs B Vancampfort D Veronese N Solmi M Gaughran F Manu P. The prevalence and predictors of obstructive sleep apnea in major depressive disorder, bipolar disorder and schizophrenia: a systematic review and meta-analysis. J Affect Disord. (2016) 197:259–67. 10.1016/j.jad.2016.02.060 26999550

[17] Berk M Williams LJ Jacka FN O'Neil A Pasco JA Moylan S. So depression is an inflammatory disease, but where does the inflammation come from? BMC Med. (2013) 11:200. 10.1186/1741-7015-11-200 24228900 PMC3846682

[18] Pan M-L Tsao H-M Hsu C-C Wu K-M Hsu T-S Wu Y-T. Bidirectional association between obstructive sleep apnea and depression: a population-based longitudinal study. Medicine. (2016) 95:e4833–e. 10.1097/MD.0000000000004833 27631236 PMC5402579

[19] Weiner CL Meredith Elkins R Pincus D Comer J. Anxiety sensitivity and sleep-related problems in anxious youth. J Anxiety Disord. (2015) 32:66–72. 10.1016/j.janxdis.2015.03.009 25863826 PMC5340315

[20] Tiller JWG. Depression and anxiety. Med J Aust. (2013) 199:S28–31. 10.5694/mja12.10628 25370281

[21] Olaya Guzmán B Essau CA. Life events. In: Goldstein S, Naglieri JA, editors. Encyclopedia of Child Behavior and Development. Boston, MA: Springer (2011) 885–7. 10.1007/978-0-387-79061-9_1656

[22] Karatzias T Yan E Jowett S. Adverse life events and health: a population study in Hong Kong. J Psychosom Res. (2015) 78:173–7. 10.1016/j.jpsychores.2014.11.024 25498319

[23] Tripathi A Bagchi S Singh J Pandey P Tripathi S Gupta NK. Lifestyle and occupational stress: a potential risk factor for obstructive sleep apnea in nonobese male subjects. J Prosthodont. (2018) 27:716–21. 10.1111/jopr.12627 28833858

[24] Deng H Guo P Zheng M Huang J Xue Y Zhan X. Epidemiological characteristics of atrial fibrillation in Southern China: results from the Guangzhou heart study. Sci Rep. (2018) 8:17829. 10.1038/s41598-018-35928-w 30546024 PMC6292893

[25] Duan X Zheng M He S Lao L Huang J Zhao W. Association between physical activity and risk of obstructive sleep apnea. Sleep Breath. (2021) 25:1925–34. 10.1007/s11325-021-02318-y 33585989

[26] Netzer NC Stoohs RA Netzer CM Clark K Strohl KP. Using the Berlin questionnaire to identify patients at risk for the sleep apnea syndrome. Ann Intern Med. (1999) 131:485–91. 10.7326/0003-4819-131-7-199910050-00002 10507956

[27] Tan A Yin JD Tan LW van Dam RM Cheung YY Lee CH. Using the Berlin questionnaire to predict obstructive sleep apnea in the general population. J Clin Sleep Med. (2017) 13:427–32. 10.5664/jcsm.6496 27855742 PMC5337590

[28] Radloff LS. The Ces-D scale: a self-report depression scale for research in the general population. Appl Psychol Meas. (1977) 1:385–401. 10.1177/01466216770010030623302475

[29] Zung WW. A rating instrument for anxiety disorders. Psychosomatics. (1971) 12:371–9. 10.1016/S0033-3182(71)71479-0 5172928

[30] Stahl D Sum CF Lum SS Liow PH Chan YH Verma S. Screening for depressive symptoms: validation of the center for epidemiologic studies depression scale (Ces-D) in a multiethnic group of patients with diabetes in Singapore. Diabetes Care. (2008) 31:1118–9. 10.2337/dc07-2019 18337303

[31] Moon JR Huh J Song J Kang IS Park SW Chang SA. The center for epidemiologic studies depression scale is an adequate screening instrument for depression and anxiety disorder in adults with congential heart disease. Health Qual Life Outcomes. (2017) 15:176. 10.1186/s12955-017-0747-0 28874154 PMC5585982

[32] Dunstan DA Scott N Todd AK. Screening for anxiety and depression: reassessing the utility of the zung scales. BMC Psychiatry. (2017) 17:329. 10.1186/s12888-017-1489-6 28886698 PMC5591521

[33] Gorman JM. Comorbid depression and anxiety spectrum disorders. Depress Anxiety. (1996) 4:160–8. 10.1002/(SICI)1520-6394(1996)4:4 <160::AID-DA2>3.0.CO;2-J 9166648

[34] Hirschfeld RMA. The comorbidity of major depression and anxiety disorders: recognition and management in primary care. Prim Care Companion J Clin Psychiatry. (2001) 3:244–54. 10.4088/PCC.v03n0609 15014592 PMC181193

[35] Kornerup H Osler M Boysen G Barefoot J Schnohr P Prescott E. Major life events increase the risk of stroke but not of myocardial infarction: results from the Copenhagen city heart study. Eur J Cardiovasc Prev Rehabil. (2010) 17:113–8. 10.1097/HJR.0b013e3283359c18 20038841 PMC3634577

[36] Salleh MR. Life event, stress and illness. Malays J Med Sci. (2008) 15:9–18. 22589633 PMC3341916

[37] Lehto SM Sahlman J Soini EJ Gylling H Vanninen E Seppä J. The association between anxiety and the degree of illness in mild obstructive sleep apnoea. Clin Respir J. (2013) 7:197–203. 10.1111/j.1752-699X.2012.00304.x 22686135

[38] Macey KE Macey PM Woo MA Henderson LA Frysinger RC Harper RK. Inspiratory loading elicits aberrant FMRI signal changes in obstructive sleep apnea. Respir Physiol Neurobiol. (2006) 151:44–60. 10.1016/j.resp.2005.05.024 15993658

[39] Sapolsky RM Uno H Rebert CS Finch CE. Hippocampal damage associated with prolonged glucocorticoid exposure in primates. J Neurosci. (1990) 10:2897–902. 10.1523/JNEUROSCI.10-09-02897.1990 2398367 PMC6570248

[40] Kumar R Macey PM Cross RL Woo MA Yan-Go FL Harper RM. Neural alterations associated with anxiety symptoms in obstructive sleep apnea syndrome. Depress Anxiety. (2009) 26:480–91. 10.1002/da.20531 18828142 PMC4041684

[41] Sheline YI. Neuroimaging studies of mood disorder effects on the brain. Biol Psychiatry. (2003) 54:338–52. 10.1016/S0006-3223(03)00347-0 12893109

[42] Owen JE BenediktsdÓttir B Gislason T Robinson SR. Neuropathological investigation of cell layer thickness and myelination in the hippocampus of people with obstructive sleep apnea. Sleep. (2019) 42:1–13. 10.1093/sleep/zsy199 30346595

[43] Naudé PJW Roest AM Stein DJ de Jonge P Doornbos B. Anxiety disorders and crp in a population cohort study with 54,326 participants: the lifelines study. World J Biol Psychiatry. (2018) 19:461–70. 10.1080/15622975.2018.1433325 29376460

[44] Baril AA Carrier J Lafreniere A Warby S Poirier J Osorio RS. Biomarkers of dementia in obstructive sleep apnea. Sleep Med Rev. (2018) 42:139–48. 10.1016/j.smrv.2018.08.001 30241998 PMC8803351

[45] Khan AM Ashizawa S Hlebowicz V Appel DW. Anemia of aging and obstructive sleep apnea. Sleep Breath. (2011) 15:29–34. 10.1007/s11325-010-0326-7 20162370

[46] Park S-Y Nam H-W. Aging and obstructive sleep apnea syndrome. J Korean Sleep Res Soc. (2011) 8:1–3. 10.13078/jksrs.11001

[47] You H Teng M Gao CX Yang B Hu S Wang T. Construction of a nomogram for predicting survival in elderly patients with lung adenocarcinoma: a retrospective cohort study. Front Med. (2021) 8:680679. 10.3389/fmed.2021.680679 34336886 PMC8316725

[48] Schröder CM O'Hara R. Depression and obstructive sleep apnea (Osa). Ann Gen Psychiatry. (2005) 4:13. 10.1186/1744-859X-4-13 15982424 PMC1181621

[49] Theorell-Haglöw J Åkerstedt T Schwarz J Lindberg E. Predictors for development of excessive daytime sleepiness in women: a population-based 10-year follow-up. Sleep. (2015) 38:1995–2003. 10.5665/sleep.5258 26237774 PMC4667375

[50] Gould CE Spira AP Liou-Johnson V Cassidy-Eagle E Kawai M Mashal N. Association of anxiety symptom clusters with sleep quality and daytime sleepiness. J Gerontol Series B Psychol Sci Soc Sci. (2018) 73:413–20. 10.1093/geronb/gbx020 28379498 PMC6074813

[51] Liu Y Peng T Zhang S Tang K. The relationship between depression, daytime napping, daytime dysfunction, and snoring in 05 million Chinese populations: exploring the effects of socio-economic status and age. BMC Public Health. (2018) 18:759. 10.1186/s12889-018-5629-9 29914433 PMC6007059

[52] Vahtera J Kivimäki M Hublin C Korkeila K Suominen S Paunio T. Liability to anxiety and severe life events as predictors of new-onset sleep disturbances. Sleep. (2007) 30:1537–46. 10.1093/sleep/30.11.1537 18041486 PMC2082106

[53] Cairney J Krause N. Negative life events and age-related decline in mastery: are older adults more vulnerable to the control-eroding effect of stress? J Gerontol Series B. (2008) 63:S162–70. 10.1093/geronb/63.3.S162 18559691

[54] World Health Organization. Aging and Health. (2018). Available online at: https://www.who.int/news-room/fact-sheets/detail/ageing-and-health (accessed October 4, 2021).

[55] World Health O. Integrated Care for Older People: Guidelines on Community-Level Interventions to Manage Declines in Intrinsic Capacity. Geneva: World Health Organization (2017). 29608259

[56] Morley JE Sanford A Bourey R. Sleep apnea: a geriatric syndrome. J Am Med Dir Assoc. (2017) 18:899–904. 10.1016/j.jamda.2017.08.020 29080571

[57] Huang Y Wang Y Wang H Liu Z Yu X Yan J. Prevalence of mental disorders in China: a cross-sectional epidemiological study. Lancet Psychiatry. (2019) 6:211–24. 10.1016/S2215-0366(18)30511-X 30792114

