# Supplementary material for: Associations of Depression, Anxiety, and Life Events With the Risk of Obstructive Sleep Apnea Evaluated by Berlin Questionnaire
Source: Front Med (Lausanne). 2022 Apr 7;9:799792. doi: 10.3389/fmed.2022.799792 (PMC9021543; doi:10.3389/fmed.2022.799792)
Supplement: Supplementary file 1 [file Data_Sheet_1.docx]

**Supplementary material**

**Figure S1.** The Life Event Questionnaire.

**Figure S2.** Directed acyclic graph (DAG) model of OSA.

**Table S1.** Sensitivity analysis on the association of OSA risk with depression, anxiety, and life events by excluding participants aged 60 years or more

**Table S2.** Sensitivity analysis on the association of OSA risk with CES-D score, and SAS index score by excluding participants aged 60 years or more

**Table S4.** Sensitivity analysis on the association of OSA risk with CES-D score and SAS index score by excluding participants with cardiovascular diseases

**Table S5.** Association of depression, anxiety, and life events with snoring risk

**Table S6.** Association of depression, anxiety, and life events with daytime sleepiness risk

**Table S7** Association of depression, anxiety, and life events with obesity risk

**Table S8** Association of depression, anxiety, and life events with hypertension risk

**
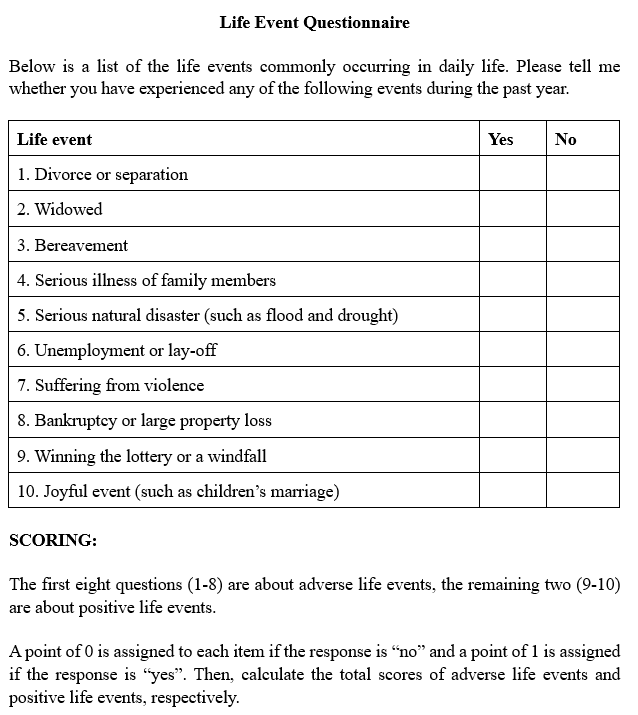
**

**Figure S1. The Life Event Questionnaire.**


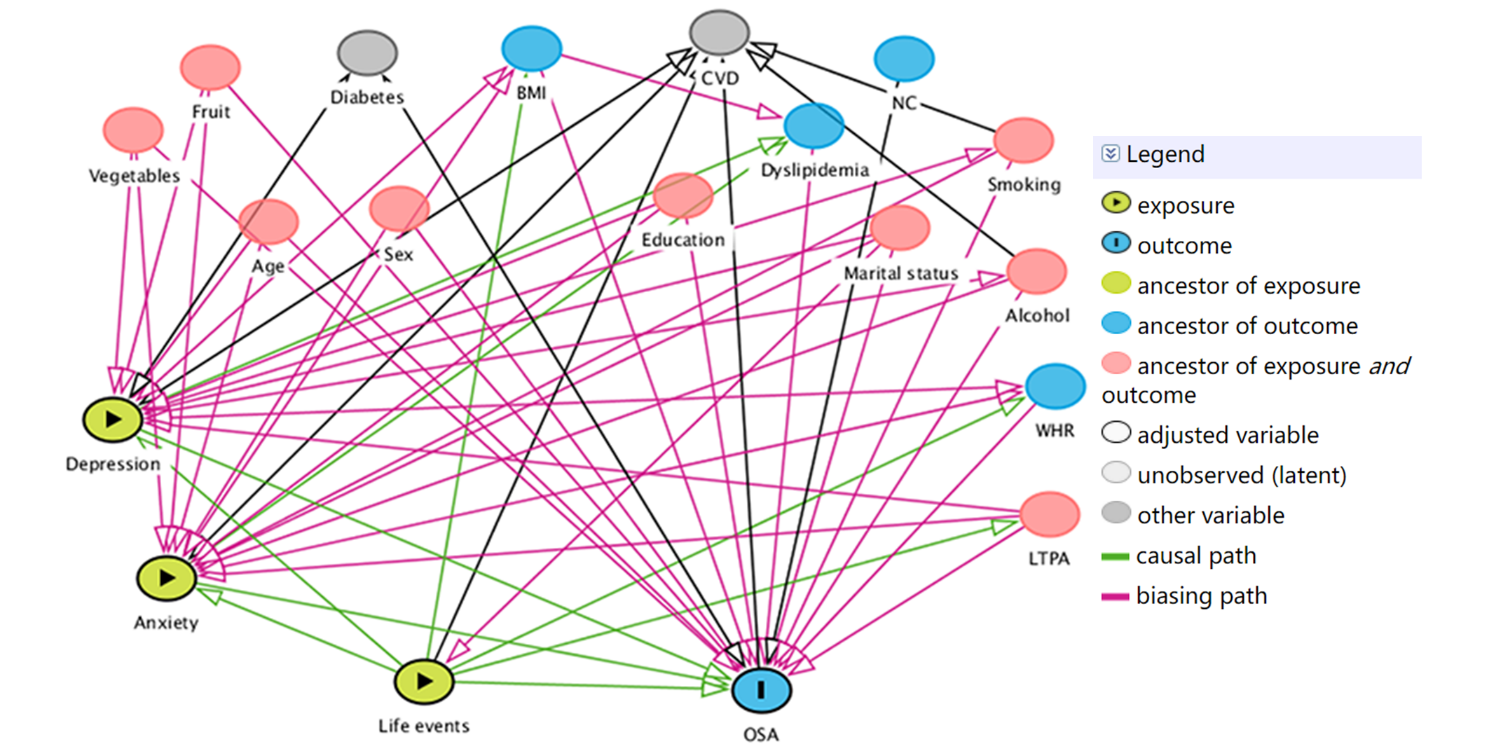


Figure S2. Directed acyclic graph (DAG) model of OSA.

BMI represents body mass index; NC represents neck circumference; WHR represents waist-hip ratio; LTPA represents leisure-time physical activity; OSA represents obstructive sleep apnea.

Table S1 Sensitivity analysis on the association of OSA risk with depression, anxiety, and life events by excluding participants aged 60 years or more

|  | N^a^ | |  | Effect estimates | | |
| --- | --- | --- | --- | --- | --- | --- |
|  | Non-OSA group | OSA group |  | Unadjusted OR (95% CI) | Adjusted OR (95% CI) ^b^ | Adjusted OR (95% CI) ^c^ |
| Depression |  |  |  |  |  |  |
| No | 5191 | 674 |  | 1.00 | 1.00 | 1.00 |
| Yes | 77 | 18 |  | 1.80 (1.04, 2.96) | 1.98 (1.11, 3.37) | 2.49 (1.36, 4.34) |
| Anxiety |  |  |  |  |  |  |
| No | 5202 | 675 |  | 1.00 | 1.00 | 1.00 |
| Yes | 66 | 17 |  | 1.99 (1.12, 3.32) | 2.42 (1.32, 4.22) | 3.81 (2.03, 6.83) |
| Combination of depression and anxiety |  |  |  |  |  |  |
| Neither | 5154 | 669 |  | 1.00 | 1.00 | 1.00 |
| One | 85 | 11 |  | 1.01 (0.50, 1.80) | 1.07 (0.53, 1.99) | 1.42 (0.69, 2.68) |
| Both | 29 | 12 |  | 3.19 (1.56, 6.12) | 4.12 (1.90, 8.41) | 6.26 (2.75, 13.55) |
| *P* for trend |  |  |  | 0.006 | 0.894 | 0.026 |
| Adverse life events |  |  |  |  |  |  |
| No | 4722 | 606 |  | 1.00 | 1.00 | 1.00 |
| Yes | 546 | 86 |  | 1.23 (0.96, 1.56) | 1.29 (0.99, 1.66) | 1.28 (0.98, 1.67) |
| Positive life events |  |  |  |  |  |  |
| No | 4877 | 625 |  | 1.00 | 1.00 | 1.00 |
| Yes | 391 | 67 |  | 1.34 (1.01, 1.74) | 1.31 (0.98, 1.73) | 1.20 (0.88, 1.62) |
| Life event score |  |  |  |  |  |  |
| <0 | 349 | 54 |  | 1.00 | 1.00 | 1.00 |
| 0 | 4410 | 562 |  | 0.82 (0.62, 1.12) | 0.82 (0.61, 1.14) | 0.91 (0.65, 1.28) |
| >0 | 509 | 76 |  | 0.97 (0.67, 1.41) | 1.02 (0.69, 1.51) | 1.12 (0.74, 1.71) |
| *P* for trend |  |  |  | 0.931 | <0.001 | <0.001 |
| Every 1-unit increment |  |  |  | 1.01 (0.84, 1.19) | 1.03 (0.86, 1.24) | 1.05 (0.87, 1.27) |

^a^ N represents sample size for the non-OSA group or for the OSA group; OSA represents obstructive sleep apnea.

^b^ Adjustment for age, sex, education, marital status, waist-hip ratio, leisure-time physical activity, smoking, alcohol drinking, fruit intake, vegetables intake, and dyslipidemia.

^c^ Additional adjustment for body mass index.

Table S2 Sensitivity analysis on the association of OSA risk with CES-D score and SAS index score by excluding participants aged 60 years or more

|  | N^a^ | |  | Effect estimates | | |
| --- | --- | --- | --- | --- | --- | --- |
|  | Non-OSA group | OSA group |  | Unadjusted OR (95% CI) | Adjusted OR (95% CI) ^b^ | Adjusted OR (95% CI) ^c^ |
| CES-D score |  |  |  |  |  |  |
| Tertile 1 | 2176 | 294 |  | 1.00 | 1.00 | 1.00 |
| Tertile 2 | 1403 | 168 |  | 0.89 (0.72, 1.08) | 1.01 (0.81, 1.24) | 1.11 (0.89, 1.39) |
| Tertile 3 | 1689 | 230 |  | 1.01 (0.84, 1.21) | 1.09 (0.90, 1.32) | 1.18 (0.96, 1.45) |
| *P* for trend |  |  |  | 0.999 | <0.001 | <0.001 |
| Every 1-unit increment |  |  |  | 1.13 (1.10, 1.15) | 1.13 (1.11, 1.15) | 1.13 (1.10, 1.15) |
| SAS index score |  |  |  |  |  |  |
| Tertile 1 | 2133 | 255 |  | 1.00 | 1.00 | 1.00 |
| Tertile 2 | 1547 | 213 |  | 1.15 (0.95, 1.40) | 1.20 (0.98, 1.46) | 1.24 (1.01, 1.54) |
| Tertile 3 | 1588 | 224 |  | 1.18 (0.97, 1.43) | 1.32 (1.08, 1.61) | 1.43 (1.15, 1.77) |
| *P* for trend |  |  |  | 0.082 | <0.001 | <0.001 |
| Every 1-unit increment |  |  |  | 1.03 (1.01, 1.04) | 1.04 (1.02, 1.06) | 1.05 (1.04, 1.07) |

^a^ N represents sample size for the non-OSA group or for the OSA group; OSA represents obstructive sleep apnea; CES-D score represents the Center for Epidemiologic Studies Depression Scale score; SAS index score represents index score from Zung’s self-rating anxiety scale.

^b^ Adjustment for age, sex, education, marital status, waist-hip ratio, leisure-time physical activity, smoking, alcohol drinking, fruit intake, vegetables intake, and dyslipidemia.

^c^ Additional adjustment for body mass index.

Table S3 Sensitivity analysis on the association of OSA risk with depression, anxiety, and life events by excluding participants with cardiovascular diseases

|  | N^a^ | |  | Effect estimates | | |
| --- | --- | --- | --- | --- | --- | --- |
|  | Non-OSA group | OSA group |  | Unadjusted OR (95% CI) | Adjusted OR (95% CI) ^b^ | Adjusted OR (95% CI) ^c^ |
| Depression |  |  |  |  |  |  |
| No | 8364 | 1233 |  | 1.00 | 1.00 | 1.00 |
| Yes | 115 | 21 |  | 1.24 (0.75, 1.94) | 1.42 (0.85, 2.27) | 1.64 (0.96, 2.67) |
| Anxiety |  |  |  |  |  |  |
| No | 8381 | 1232 |  | 1.00 | 1.00 | 1.00 |
| Yes | 98 | 22 |  | 1.53 (0.93, 2.39) | 2.01 (1.21, 3.21) | 2.47 (1.45, 4.05) |
| Combination of depression and anxiety |  |  |  |  |  |  |
| Neither | 8310 | 1224 |  | 1.00 | 1.00 | 1.00 |
| One | 125 | 17 |  | 0.92 (0.53, 1.49) | 1.50 (0.66, 1.88) | 1.31 (0.74, 2.19) |
| Both | 44 | 13 |  | 2.01 (1.03, 3.63) | 2.45 (1.22, 4.61) | 3.10 (1.48, 6.07) |
| *P* for trend |  |  |  | 0.119 | 0.136 | <0.001 |
| Adverse life events |  |  |  |  |  |  |
| No | 7667 | 1136 |  | 1.00 | 1.00 | 1.00 |
| Yes | 812 | 118 |  | 0.98 (0.80, 1.20) | 1.07 (0.86, 1.32) | 1.08 (0.86, 1.34) |
| Positive life events |  |  |  |  |  |  |
| No | 7848 | 1141 |  | 1.00 | 1.00 | 1.00 |
| Yes | 631 | 113 |  | 1.23 (0.99, 1.51) | 1.27 (1.02, 1.57) | 1.23 (0.98, 1.54) |
| Life event score |  |  |  |  |  |  |
| <0 | 568 | 96 |  | 1.00 | 1.00 | 1.00 |
| 0 | 7152 | 1053 |  | 0.87 (0.70, 1.10) | 0.84 (0.67, 1.06) | 0.86 (0.68, 1.11) |
| >0 | 759 | 105 |  | 0.82 (0.61, 1.10) | 0.86 (0.63, 1.17) | 0.90 (0.65, 1.24) |
| *P* for trend |  |  |  | 0.200 | <0.001 | <0.001 |
| Every 1-unit increment |  |  |  | 0.92 (0.80, 1.06) | 0.95 (0.83, 1.10) | 0.96 (0.83, 1.12) |

^a^ N represents sample size for the non-OSA group or for the OSA group; OSA represents obstructive sleep apnea.

^b^ Adjustment for age, sex, education, marital status, waist-hip ratio, leisure-time physical activity, smoking, alcohol drinking, fruit intake, vegetables intake, and dyslipidemia.

^c^ Additional adjustment for body mass index.

Table S4 Sensitivity analysis on the association of OSA risk with CES-D score and SAS index score by excluding participants with cardiovascular diseases

|  | N^a^ | |  | Effect estimates | | |
| --- | --- | --- | --- | --- | --- | --- |
|  | Non-OSA group | OSA group |  | Unadjusted OR (95% CI) | Adjusted OR (95% CI) ^b^ | Adjusted OR (95% CI) ^c^ |
| CES-D score |  |  |  |  |  |  |
| Tertile 1 | 3528 | 520 |  | 1.00 | 1.00 | 1.00 |
| Tertile 2 | 2350 | 331 |  | 0.96 (0.82, 1.11) | 1.06 (0.91, 1.23) | 1.16 (0.99, 1.36) |
| Tertile 3 | 2601 | 403 |  | 1.05 (0.91, 1.21) | 1.16 (1.01, 1.34) | 1.28 (1.10, 1.49) |
| *P* for trend |  |  |  | 0.530 | <0.001 | <0.001 |
| Every 1-unit increment |  |  |  | 1.12 (1.10, 1.14) | 1.12 (1.10, 1.14) | 1.12 (1.10, 1.14) |
| SAS index score |  |  |  |  |  |  |
| Tertile 1 | 3426 | 474 |  | 1.00 | 1.00 | 1.00 |
| Tertile 2 | 2481 | 371 |  | 1.08 (0.93, 1.25) | 1.15 (0.99, 1.33) | 1.19 (1.01, 1.39) |
| Tertile 3 | 2572 | 409 |  | 1.15 (1.00, 1.32) | 1.29 (1.11, 1.49) | 1.40 (1.20, 1.64) |
| *P* for trend |  |  |  | 0.053 | <0.001 | <0.001 |
| Every 1-unit increment |  |  |  | 1.02 (1.01, 1.03) | 1.03 (1.02, 1.05) | 1.04 (1.03, 1.06) |

^a^ N represents sample size for the non-OSA group or for the OSA group; OSA represents obstructive sleep apnea; CES-D score represents the Center for Epidemiologic Studies Depression Scale score; SAS index score represents index score from Zung’s self-rating anxiety scale.

^b^ Adjustment for age, sex, education, marital status, waist-hip ratio, leisure-time physical activity, smoking, alcohol drinking, fruit intake, vegetables intake, and dyslipidemia.

^c^ Additional adjustment for body mass index.

Table S5 Association of depression, anxiety, and life events with snoring risk

|  | N^a^ | |  | Effect estimates | | |
| --- | --- | --- | --- | --- | --- | --- |
|  | Negative group | Positive group |  | Unadjusted OR (95% CI) | Adjusted OR (95% CI) ^b^ | Adjusted OR (95% CI) ^c^ |
| Depression |  |  |  |  |  |  |
| No | 7155 | 2979 |  | 1.00 | 1.00 | 1.00 |
| Yes | 99 | 54 |  | 1.31 (0.93, 1.82) | 1.47 (1.03, 2.08) | 1.48 (1.03, 2.12) |
| Every 1-unit increment of CES-D score |  |  |  | 1.01 (1.00, 1.02) | 1.02 (1.01, 1.03) | 1.02 (1.01, 1.04) |
| Anxiety |  |  |  |  |  |  |
| No | 7158 | 2986 |  | 1.00 | 1.00 | 1.00 |
| Yes | 96 | 47 |  | 1.17 (0.82, 1.66) | 1.40 (0.97, 2.01) | 1.37 (0.93, 1.99) |
| Every 1-unit increment of SAS index score |  |  |  | 1.01 (1.00, 1.02) | 1.02 (1.01, 1.03) | 1.02 (1.01, 1.03) |
| Combination of depression and anxiety |  |  |  |  |  |  |
| Neither | 7101 | 2960 |  | 1.00 | 1.00 | 1.00 |
| One | 111 | 45 |  | 0.97 (0.68, 1.37) | 1.17 (0.81, 1.66) | 1.15 (0.79, 1.66) |
| Both | 42 | 28 |  | 1.60 (0.98, 2.57) | 1.80 (1.08, 2.96) | 1.79 (1.05, 3.00) |
| *P* for trend |  |  |  | 0.146 | <0.001 | <0.001 |
| Adverse life events |  |  |  |  |  |  |
| No | 6568 | 2730 |  | 1.00 | 1.00 | 1.00 |
| Yes | 686 | 303 |  | 1.06 (0.92, 1.22) | 1.11 (0.95, 1.28) | 1.10 (0.94, 1.28) |
| Positive life events |  |  |  |  |  |  |
| No | 6736 | 2772 |  | 1.00 | 1.00 | 1.00 |
| Yes | 518 | 261 |  | 1.22 (1.05, 1.43) | 1.28 (1.08, 1.50) | 1.26 (1.07, 1.49) |
| Every 1-unit increment of life event score |  |  |  | 0.97 (0.88, 1.07) | 0.98 (0.88, 1.08) | 0.97 (0.88, 1.08) |

^a^ N represents sample size for the non-OSA group or for the OSA group; OSA represents obstructive sleep apnea; CES-D score represents the Center for Epidemiologic Studies Depression Scale score; SAS index score represents index score from Zung’s self-rating anxiety scale.

^b^ Adjustment for age, sex, education, marital status, waist-hip ratio, leisure-time physical activity, smoking, alcohol drinking, fruit intake, vegetables intake, and dyslipidemia.

^c^ Additional adjustment for daytime sleepiness, body mass index and hypertension.

Table S6 Association of depression, anxiety, and life events with daytime sleepiness risk

|  | N^a^ | |  | Effect estimates | | |
| --- | --- | --- | --- | --- | --- | --- |
|  | Negative group | Positive group |  | Unadjusted OR (95% CI) | Adjusted OR (95% CI) ^b^ | Adjusted OR (95% CI) ^c^ |
| Depression |  |  |  |  |  |  |
| No | 9694 | 440 |  | 1.00 | 1.00 | 1.00 |
| Yes | 120 | 33 |  | 6.06 (4.02, 8.90) | 5.95 (3.93, 8.79) | 5.70 (3.75, 8.44) |
| Every 1-unit increment of CES-D score |  |  |  | 1.13 (1.11, 1.14) | 1.13 (1.11, 1.15) | 1.13 (1.11, 1.15) |
| Anxiety |  |  |  |  |  |  |
| No | 9710 | 434 |  | 1.00 | 1.00 | 1.00 |
| Yes | 104 | 39 |  | 8.39 (5.67, 12.17) | 8.38 (5.65, 12.21) | 8.10 (5.44, 11.84) |
| Every 1-unit increment of SAS index score |  |  |  | 1.15 (1.13, 1.16) | 1.15 (1.14, 1.17) | 1.15 (1.13, 1.17) |
| Combination of depression and anxiety |  |  |  |  |  |  |
| Neither | 9640 | 421 |  | 1.00 | 1.00 | 1.00 |
| One | 124 | 32 |  | 5.91 (3.90, 8.71) | 5.79 (3.81, 8.57) | 5.62 (3.69, 8.33) |
| Both | 50 | 20 |  | 9.16 (5.29, 15.28) | 9.20 (5.28, 15.46) | 8.78 (5.02, 14.82) |
| *P* for trend |  |  |  | <0.001 | 0.375 | 0.748 |
| Adverse life events |  |  |  |  |  |  |
| No | 8885 | 413 |  | 1.00 | 1.00 | 1.00 |
| Yes | 929 | 60 |  | 1.39 (1.04, 1.82) | 1.35 (1.01, 1.77) | 1.34 (1.10 1.76) |
| Positive life events |  |  |  |  |  |  |
| No | 9065 | 443 |  | 1.00 | 1.00 | 1.00 |
| Yes | 749 | 30 |  | 0.82 (0.55, 1.17) | 0.83 (0.56, 1.19) | 0.82 (0.55, 1.18) |
| Every 1-unit increment of life event score |  |  |  | 1.33 (1.09, 1.61) | 1.31 (1.07, 1.59) | 1.31 (1.07, 1.58) |

^a^ N represents sample size for the non-OSA group or for the OSA group; OSA represents obstructive sleep apnea; CES-D score represents the Center for Epidemiologic Studies Depression Scale score; SAS index score represents index score from Zung’s self-rating anxiety scale.

^b^ Adjustment for age, sex, education, marital status, waist-hip ratio, leisure-time physical activity, smoking, alcohol drinking, fruit intake, vegetables intake, and dyslipidemia.

^c^ Additional adjustment for snoring, body mass index and hypertension.

Table S7 Association of depression, anxiety, and life events with obesity risk

|  | N^a^ | |  | Effect estimates | | |
| --- | --- | --- | --- | --- | --- | --- |
|  | Non-obesity group | Obesity group |  | Unadjusted OR (95% CI) | Adjusted OR (95% CI) ^b^ | Adjusted OR (95% CI) ^c^ |
| Depression |  |  |  |  |  |  |
| No | 9606 | 528 |  | 1.00 | 1.00 | 1.00 |
| Yes | 147 | 6 |  | 0.74 (0.29, 1.55) | 0.75 (0.29, 1.60) | 0.68 (0.26, 1.48) |
| Every 1-unit increment of CES-D score |  |  |  | 0.95 (0.92, 0.98) | 0.96 (0.93, 0.98) | 0.95 (0.92, 0.98) |
| Anxiety |  |  |  |  |  |  |
| No | 9618 | 526 |  | 1.00 | 1.00 | 1.00 |
| Yes | 135 | 8 |  | 1.08 (0.49, 2.08) | 1.28 (0.56, 2.52) | 1.11 (0.48, 2.26) |
| Every 1-unit increment of SAS index score |  |  |  | 0.99 (0.97, 1.01) | 0.99 (0.97, 1.01) | 0.98 (0.96, 1.01) |
| Combination of depression and anxiety |  |  |  |  |  |  |
| Neither | 9537 | 524 |  | 1.00 | 1.00 | 1.00 |
| One | 150 | 6 |  | 0.73 (0.28, 1.51) | 0.83 (0.32, 1.75) | 0.83 (0.32, 1.80) |
| Both | 66 | 4 |  | 1.10 (0.33, 2.68) | 1.15 (0.34, 2.94) | 0.90 (0.26, 2.39) |
| *P* for trend |  |  |  | 0.766 | <0.001 | <0.001 |
| Adverse life events |  |  |  |  |  |  |
| No | 8807 | 491 |  | 1.00 | 1.00 | 1.00 |
| Yes | 946 | 43 |  | 0.82 (0.58, 1.11) | 0.88 (0.62, 1.20) | 0.84 (0.59, 1.16) |
| Positive life events |  |  |  |  |  |  |
| No | 9016 | 492 |  | 1.00 | 1.00 | 1.00 |
| Yes | 737 | 42 |  | 1.04 (0.74, 1.43) | 1.06 (0.75, 1.47) | 0.98 (0.69, 1.36) |
| Every 1-unit increment of life event score |  |  |  | 0.92 (0.75, 1.12) | 0.94 (0.76, 1.16) | 0.95 (0.77, 1.17) |

^a^ N represents sample size for the non-OSA group or for the OSA group; OSA represents obstructive sleep apnea; CES-D score represents the Center for Epidemiologic Studies Depression Scale score; SAS index score represents index score from Zung’s self-rating anxiety scale.

^b^ Adjustment for age, sex, education, marital status, waist-hip ratio, leisure-time physical activity, smoking, alcohol drinking, fruit intake, vegetables intake, and dyslipidemia.

^c^ Additional adjustment for snoring, daytime sleepiness and hypertension of Berlin Questionnaire.

Table S8 Association of depression, anxiety, and life events with hypertension risk

|  | N^a^ | |  | Effect estimates | | |
| --- | --- | --- | --- | --- | --- | --- |
|  | Non-hypertension group | Hypertension group |  | Unadjusted OR (95% CI) | Adjusted OR (95% CI) ^b^ | Adjusted OR (95% CI) ^c^ |
| Depression |  |  |  |  |  |  |
| No | 7149 | 2985 |  | 1.00 | 1.00 | 1.00 |
| Yes | 115 | 38 |  | 0.79 (0.54, 1.13) | 0.87 (0.58, 1.26) | 0.96 (0.64, 1.42) |
| Every 1-unit increment of CES-D score |  |  |  | 0.98 (0.96, 0.99) | 0.98 (0.97, 0.99) | 0.99 (0.97, 1.01) |
| Anxiety |  |  |  |  |  |  |
| No | 7159 | 2985 |  | 1.00 | 1.00 | 1.00 |
| Yes | 105 | 38 |  | 0.87 (0.59, 1.25) | 0.99 (0.66, 1.45) | 1.13 (0.75, 1.67) |
| Every 1-unit increment of SAS  index score |  |  |  | 0.98 (0.97, 0.99) | 0.98 (0.97, 0.99) | 0.99 (0.98, 1.01) |
| Combination of depression and anxiety |  |  |  |  |  |  |
| Neither | 7093 | 2968 |  | 1.00 | 1.00 | 1.00 |
| One | 122 | 34 |  | 0.67 (0.45, 0.96) | 0.74 (0.49, 1.09) | 0.82 (0.54, 1.21) |
| Both | 49 | 21 |  | 1.02 (0.60, 1.69) | 1.15 (0.66, 1.94) | 1.33 (0.75, 2.28) |
| *P* for trend |  |  |  | 0.244 | <0.001 | <0.001 |
| Adverse life events |  |  |  |  |  |  |
| No | 6559 | 2739 |  | 1.00 | 1.00 | 1.00 |
| Yes | 705 | 284 |  | 0.96 (0.83, 1.11) | 1.10 (0.94, 1.28) | 1.11 (0.95, 1.29) |
| Positive life events |  |  |  |  |  |  |
| No | 6730 | 2778 |  | 1.00 | 1.00 | 1.00 |
| Yes | 534 | 245 |  | 1.11 (0.95, 1.30) | 1.14 (0.96, 1.34) | 1.11 (0.94, 1.31) |
| Every 1-unit increment of life event score |  |  |  | 0.93 (0.85, 1.03) | 0.99 (0.89, 1.10) | 1.01 (0.90, 1.11) |

^a^ N represents sample size for the non-OSA group or for the OSA group; OSA represents obstructive sleep apnea; CES-D score represents the Center for Epidemiologic Studies Depression Scale score; SAS index score represents index score from Zung’s self-rating anxiety scale.

^b^ Adjustment for age, sex, education, marital status, waist-hip ratio, leisure-time physical activity, smoking, alcohol drinking, fruit intake, vegetables intake, and dyslipidemia.

^c^ Additional adjustment for snoring, daytime sleepiness, and BMI of Berlin Questionnaire.
